# Usefulness of the illustrated patient-reported outcome tool ‘Okomarigoto Sheet’ for symptom expression and communication in patients with rheumatoid arthritis

**DOI:** 10.1016/j.ero.2026.03.020

**Published:** 2026-04-28

**Authors:** Mie Fusama, Hideko Nakahara, Megumi Okada, Kayoko Sakagami, Ikuyo Noguchi, Harumi Matsumura, Hiroaki Ito, Kosaku Oda, Yoshitaka Shinto, Kenshi Higami, Satomi Higami, Tetsuya Tomita

**Affiliations:** 1 Health Sciences Department of Nursing, Kansai University of International Studies, Miki, Japan; 2 Faculty of Health Science, Osaka Yukioka College of Health Science, Ibaraki, Japan; 3 Shinto Orthopaedics and Rheumatology Clinic, Osaka, Japan; 4 Kinshukai Infusion Clinic, Osaka, Japan; 5 Oda Orthopedic Surgery Rheumatology Clinic, Nishinomiya, Japan; 6 Higami Clinic of Rheumatology and Diabetology, Kashihara, Japan; 7 Graduate School of Health Sciences, Morinomiya University of Medical Sciences, Osaka, Japan

## Abstract

**Objectives:**

This study aimed to evaluate whether the ‘Okomarigoto Sheet’ (OS), an illustration-based tool for patients with rheumatoid arthritis (RA), correlates with conventional measures and elicits patient symptoms.

**Methods:**

The OS includes 3 symptom scales: morning stiffness duration, and visual analogue scale (VAS) for joint pain and VAS for fatigue. Five illustrations depicting 5 different situations for each symptom were also scored (0 = none, 1 = present, and 2 = severe, respectively). Patients with RA were recruited from 4 Japanese clinics. Correlations between the OS and conventional RA-related measures were assessed using Spearman’s rank correlation.

**Results:**

Eighty patients with RA participated in this study. The total score of all 15 illustrations positively correlated with the Disease Activity Score using the Clinical Disease Activity Index and negatively with the physical and mental component scores of the Short-Form-12. Stiffness duration, pain VAS, and fatigue VAS were each positively correlated with the respective total of 5 illustrations. Among patients scoring 0 on stiffness duration, pain VAS, or fatigue VAS, a total of 10 patients showed symptoms in the corresponding illustrations. Patient global assessment (PGA) was correlated with the total score of 5 illustrations for each of the 3 symptoms, whereas evaluator global assessment (EGA) correlated only with those for stiffness and pain, not fatigue.

**Conclusions:**

The OS reflected standard measures of disease activity and quality of life. It also has the potential to clarify symptoms that are not captured by conventional measures. Furthermore, the OS suggested that fatigue may be one of the factors contributing to the discrepancy between PGA and EGA.

**WHAT IS ALREADY KNOWN ON THIS TOPIC**

• Effective communication is essential for implementing shared decision-making (SDM) between rheumatologists and their patients. However, patients often struggle to adequately convey their symptoms and feelings to their doctors. Recently in Japan, a communication tool using illustrations, known as the ‘Okomarigoto Sheet’ (OS), was developed. Correlations between the OS and conventional assessment measures, such as the Simplified Disease Activity Index and the Health Assessment Questionnaire Disability Index, have been reported.

**WHAT THIS STUDY ADDS**

• This study revealed that the OS can elicit ‘hidden symptoms’ of patients who could not be identified through conventional measures. Furthermore, the use of OS suggested that fatigue may be a factor contributing to the discrepancy between patient global assessment and evaluator global assessment.

**HOW THIS STUDY MIGHT AFFECT RESEARCH, PRACTICE OR POLICY**

• This study suggests that the OS facilitates patients with rheumatoid arthritis in expressing their symptoms more effectively, thereby enhancing physician-patient communication and ultimately contributing to the promotion of SDM.

Alt-text: Unlabelled box dummy alt text

## INTRODUCTION

Advances in medications for patients with rheumatoid arthritis (RA) have made it possible to achieve remission or low disease activity [1]. To evaluate patients' disease activity, comprehensive evaluation indices including the Disease Activity Score using the Clinical Disease Activity Index (CDAI) and are widely used, all of which include the patient's subjective evaluation via the visual analogue scale (VAS) [2].

The VAS is widely used in RA to assess patient-reported outcomes (PROs), including pain and disease activity, due to its simplicity and ease of administration. However, patient global assessment (PGA) may be influenced by noninflammatory mechanisms [3]. For instance, PGA scores may remain elevated in patients with no active joints and normal CRP levels, attributable to noninflammatory conditions such as fibromyalgia, osteoarthritis, depression, psychological distress, and other comorbidities [4]. Furthermore, PGA correlates more strongly with pain than with swelling, whereas evaluator global assessment (EGA), assessed using the physician’s VAS, more reflects swelling than pain, highlighting a fundamental discrepancy between patient and physician assessments of RA [5,6]. Additionally, pain in patients with RA can arise from both inflammatory and noninflammatory mechanisms, making its assessment challenging [7]. Therefore, the inclusion of PGA in composite disease activity measures, such as CDAI, may result in the overestimation of inflammatory remission rates, potentially leading to overtreatment [4].

Although the PGA has its challenges, the patient's perspective is vital for clinical decision-making. Shared decision-making (SDM) refers to a collaborative process in which clinicians and patients together evaluate the best available evidence to make informed treatment decisions [8]. This is particularly applicable in situations where various reasonable treatment options are available, each bearing distinct benefits and adverse effects, and the selection of the optimal course of treatment rests on individual patient priorities [9]. Many decisions in clinical practice involve balancing benefits and risks, the relative importance of which varies among patients [9], and clinical measures alone cannot adequately encompass subjective symptoms such as pain, fatigue, or emotional distress. Therefore, healthcare decisions should be guided not only by clinical evidence but also by the individual values, preferences, and experiences of each patient [10]. Incorporating the patient's perspective enables clinicians to more comprehensively understand the real-world burden of disease and adjust treatment strategies accordingly [9]. Moreover, patients who actively engage in treatment decision-making are more likely to comprehend the rationale underlying their treatment choices, expand their knowledge, and sustain engagement in disease management, ultimately resulting in improved satisfaction and treatment adherence [[9], [10], [11], [12], [13]]. Therefore, the integration of the patient's perspective into clinical decision-making through SDM is fundamental to patient-centred care, fostering self-management skills, improving treatment adherence, and optimising long-term outcomes [13].

However, a survey of rheumatologists in Japan reported that SDM is not adequately practiced [14]. A qualitative survey of rheumatology care nurses in Japan also indicated a lack of communication between patients and physicians, with some patients describing difficulties in consulting their physicians [15]. Similarly, a survey of patients with RA indicated that they sometimes have difficulty expressing their symptoms and feelings to physicians [16]. To enable effective communication and facilitate SDM, communication tools that help patients clearly express their disease state and assist physicians and other healthcare professionals in accurately understanding them, while taking into account the limitations of the VAS, were considered necessary.

**Figure 1:**
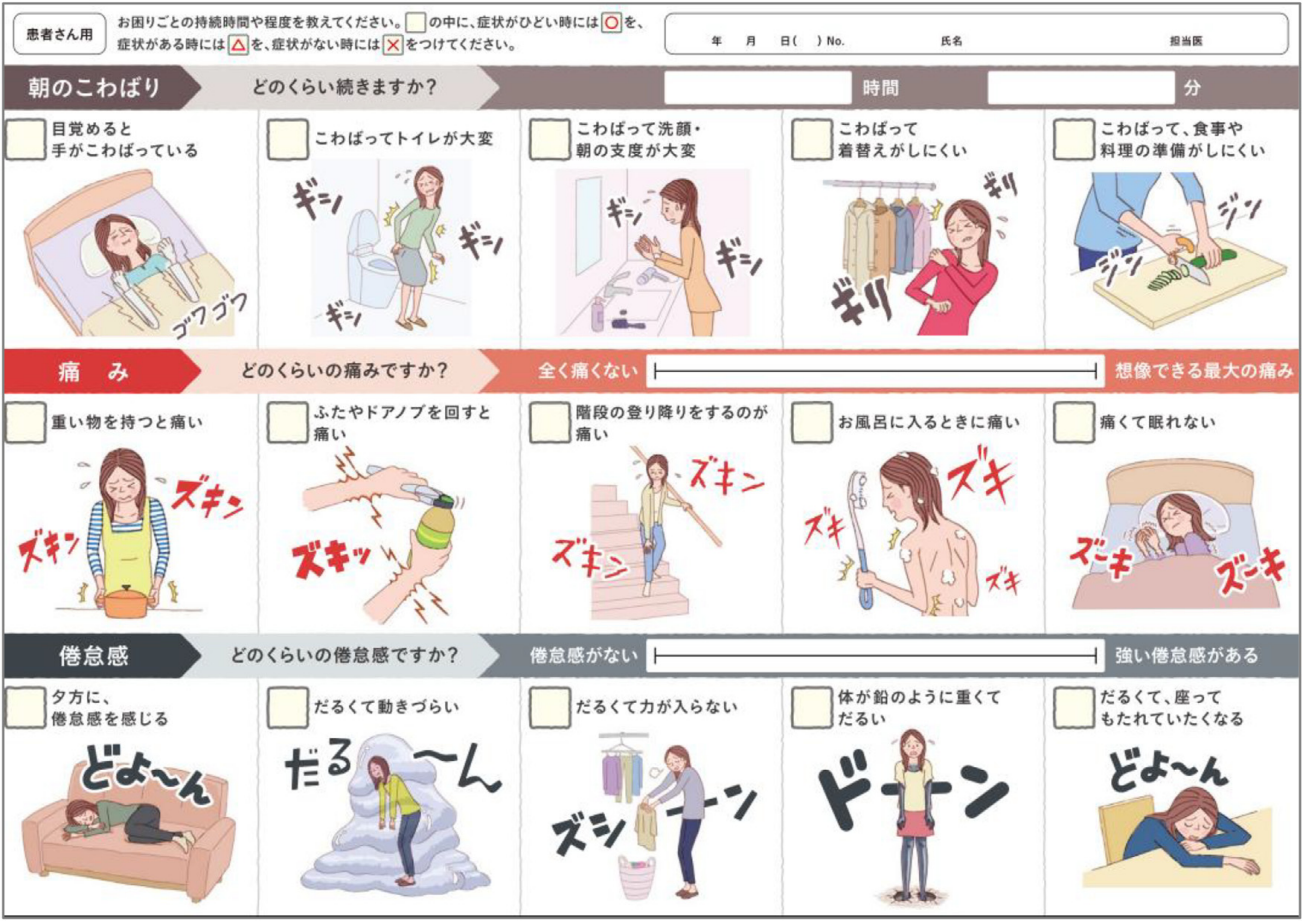
The Okomarigoto Sheet (OS). The OS consists of 3 symptom scales: morning stiffness duration, VAS for joint pain, and VAS for fatigue. The duration of morning stiffness is assessed in hours and minutes, and the VAS scales for joint pain and fatigue are assessed, respectively, as follows: 0 mm = none and 100 mm = very severe. Each of these 3 symptoms is accompanied by 5 illustrated scenarios (15 illustrations in total); each of which includes illustration-based questions. Patients were asked to mark each question with a circle (°) if the symptom was severe, a triangle (△) if the symptom was present, and a cross (×) if the symptom was absent. The detailed explanation of each illustration is as follows: The explanation shown in each of the following 5 illustrations of morning stiffness: (i) When I wake up, I feel stiffness in my hands; (ii) Due to stiffness, I find it difficult to use the toilet; (iii) Due to stiffness, I find it difficult to wash my face and get ready in the morning; (iv) Due to stiffness, I find it difficult to put on clothes; and (v) Due to stiffness, I find it difficult to prepare food. The explanation shown in each of the following 5 illustrations of joint pain: (i) Lifting heavy things is painful; (ii) Turning (things like) caps or doorknobs is painful; (iii) Climbing and descending stairs is painful; (iv) Taking a bath or shower is painful; and (v) I have trouble sleeping due to pain. The explanation shown in each of the following 5 illustrations of fatigue: (i) In the evening, I feel fatigue; (ii) It is hard to move due to fatigue; (iii) Everything seems heavy due to fatigue; (iv) My body feels as heavy as lead, and I feel sluggish; and (v) I want to sit down and lean on something due to fatigue.

Recently, Kaneko et al [17] developed a communication tool using illustrations, ‘Okomarigoto Sheet’ (OS), with other rheumatologists and patients with RA, and it has been widely used in Japan. The OS consists of 3 symptom scales-morning stiffness duration, VAS for joint pain, and VAS for fatigue-each accompanied by 5 illustrated scenarios (15 illustrations in total). These illustrations depict common daily situations associated with each symptom, allowing patients to visually assess and report symptom severity in a quantitative manner (Fig 1). This assessment tool has been reported to have a correlation with existing disease activity and physical function measures such as CDAI and the Health Assessment Questionnaire Disability Index (HAQ-DI) [18]. However, the benefits of using illustrations of the OS and the relationship between the VAS scale and the illustration assessment of the OS have not been fully evaluated.

In this study, we evaluated not only the relationship between conventional assessment tools and the OS but also the usefulness of illustrations of the OS, such as how easily patients can communicate their symptoms using the OS.

## METHODS

### Design

This was a multicentre, cross-sectional study in Japan.

### Participants

At each of the 4 participating clinics, nurses randomly invited patients to participate in the study. Patients were eligible for inclusion if they had been diagnosed with RA, were receiving treatment at 1 of the 4 participating clinics, had never used the OS, and were able to understand the purpose of the study and answer the questionnaire. Patients who had previously used the OS or who were judged by nurses to be unable to adequately understand the study and respond to the questionnaire were not included. Patients who provided informed consent were enrolled. Data collection was conducted from January to May 2025.

### OS

The OS was established through an internet-based survey involving patients with RA and certified rheumatologists, supplemented by iterative in-person interviews [17]. From March 2016, a group of 3 to 12 patients with RA representing the Japan Rheumatism Friendship Association held 6 consecutive meetings to identify the key RA symptoms they wished their physicians to better recognise. At the inaugural meeting, participants articulated their symptoms and the challenges they encountered in conveying subjective symptoms and feelings to their physicians. During the second through fifth meetings, patients assessed rough sketches of RA symptoms and selected 15 they deemed most appropriate. At the sixth meeting, refined draft illustrations were presented for patient review, and participants evaluated whether these accurately reflected their symptoms. Concurrently, 5 certified rheumatologists with 15 years of experience in RA practice convened to deliberate on strategies for enhancing patient-physician communication in clinical settings, with particular emphasis on barriers to effective communication. Subsequently, 2 patients and 6 rheumatologists gathered to review and finalise the illustrations. This sheet is presented in Figure 1, and it is currently available for download from the website of the Japan College of Rheumatology [19].

The OS consists of 3 symptom scales: morning stiffness duration, VAS for joint pain, and VAS for fatigue. The duration of morning stiffness is assessed in hours and minutes, and the VAS scales for joint pain and fatigue are assessed respectively as follows: 0 mm = none and 100 mm = very severe. Each of these 3 symptoms is accompanied by 5 illustrated scenarios (15 illustrations in total). Each Illustration provides the corresponding explanation for each situation as follows.

The explanation shown in each of the following 5 illustrations of morning stiffness: (i) When I wake up, I feel stiffness in my hands; (ii) Due to stiffness, I find it difficult to use the toilet; (iii) Due to stiffness, I find it difficult to wash my face and get ready in the morning; (iv) Due to stiffness, I find it difficult to put on clothes; and (v) Due to stiffness, I find it difficult to prepare food. The explanation shown in each of the following 5 illustrations of joint pain: (i) Lifting heavy things is painful; (ii) Turning (things like) caps or doorknobs is painful; (iii) Climbing and descending stairs is painful; (iv) Taking a bath or shower is painful; and (v) I have trouble sleeping due to pain. The explanation shown in each of the 5 illustrations of fatigue: (i) In the evening, I feel fatigue; (ii) It is hard to move due to fatigue; (iii) Everything seems heavy due to fatigue; (iv) My body feels as heavy as lead, and I feel sluggish; and (v) I want to sit down and lean on something due to fatigue.

Patients were asked to mark each illustration with a circle (°) if the symptom was severe, a triangle (△) if the symptom was present, and a cross (×) if the symptom was absent. Each illustration was scored as follows: (×); no symptom = 0, (△); present symptom = 1, (°); and severe symptom = 2. The total score of all 15 illustrations ranges from 0 to 30, indicating symptom severity. These illustrations depict common daily situations associated with each symptom, allowing patients to visually assess and report symptom severity (Fig 1). Each scale is designed to be quantitative to clarify the levels of their symptoms.

### Questionnaires

Participants were asked for their demographics, such as age and gender, and clinical characteristics. Their disease activities were assessed using CDAI [20], with swollen joint count (SJC) of 28 joints, tender joint count (TJC) of 28 joints, PGA, EGA, and serum CRP. PRO was used to evaluate functional disability with HAQ-DI [21], health-related quality of life (HL-QOL) with the 12-item Short-Form Health Survey (SF-12) [22], which consists of 3 components: the physical component summary (PCS), mental component summary (MCS), and role/social component summary (RCS). In addition, the correlations between the OS, disease activity, and HL-QOL were evaluated. The correlations between the OS and the values of PGA, EGA, SJC, and TJC were also analysed.

### Statistical analysis

#### Data analysis

Spearman’s rank correlation coefficient was used to evaluate correlation. A probability value of less than 0.05 was considered significant. All statistical analyses were conducted using EZR software, version 1.67 (Saitama Medical Center, Jichi Medical University, Japan).

#### Ethical consideration

Participants were invited to join this study voluntarily. Written informed consent was obtained from all patients. This study was approved by the Ethics Committee at Kansai University of International Studies, Japan (No. R6-21).

## RESULTS

### Patient demographics and clinical characteristics

Eighty participants responded to the questionnaire from 4 clinics. Their demographic data and clinical characteristics are presented in Table 1. The mean age was 61.4 years, and 78.8% of the participants were females. Disease activity was not high, as shown by the mean CDAI of 5.35, respectively. The mean HAQ-DI score was 0.25. Methotrexate and glucocorticoids were used in 70% and 13.8% of the patients, respectively, and 32.5% of the patients were treated with biologics.

**Table 1:** Patient demographics and clinical characteristics

Characteristics	(n = 80)
Age (y)	61.41 (12.95)
Female, n (%)	63 (78.8)
Disease duration (y)	12.58 (9.23)
SJC	1.5 (2.91)
TJC	1.03 (1.63)
EGA	9.6 (11.38)
PGA	18.73 (21.11)
CDAI	5.35 (5.92)
HAQ-DI	0.25 (0.43)
MTX use, n (%)	56 (70.0)
Glucocorticoid use, n (%)	11(13.8)
bDMARDs use, n (%)	26 (32.5)
tsDMARDs use, n (%)	11 (13.8)

Mean (Standard Deviation). CDAI, Clinical Disease Activity Index; DMARDs, disease-modifying antirheumatic drugs; EGA, evaluator global assessment; HAQ-DI, Health Assessment Questionnaire – Disability Index; MTX, methotrexate; PGA, patient global assessment; SJC, swollen joint count; TJC, total joint count; bDMARDs, biological disease-modifying antirheumatic drugs; tsDMARDs, targeted synthetic disease-modifying antirheumatic drugs.

### Correlations between the total score of all 15 illustrations, disease activity, and QOL score

The total score of all 15 illustrations was positively correlated with CDAI (*r* = 0.641) and HAQ-DI (*r* = 0.583). It was negatively correlated with PCS (*r* = −0.532) and MCS (*r* = −0.417) of SF-12, but not correlated with RCS (*r* = −0.188) (Table 2). In addition, the total scores of each of the 5 illustrations for morning stiffness, joint pain, and fatigue were also positively correlated with CDAI and HAQ, respectively (Table 2). Similarly, they were also negatively correlated with PCS and MCS of SF-12 but not correlated with RCS (Table 2). Moreover, CDAI and HAQ were also negatively correlated with PCS (*r* = −0.555 and -0.582, respectively) and MCS (*r* = −0.361 and −0.202, respectively) of SF-12, but not correlated with RCS (*r* = −0.06 and −0.183, respectively).

**Table 2:** Correlation between the total score of the illustrations, disease activity, and QOL score

	CDAI		HAQ-DI		PCS		MCS		RCS	
	*r*	*P*	*r*	*P*	*r*	*P*	*r*	*P*	*r*	*P*
Total score of whole 15 illustrations	0.641	<.001	0.583	<.001	−0.532	<.001	−0.417	<.001	−0.188	.095
Five illustrations’ total scores of MS	0.526	<.001	0.419	<.001	−0.497	<.001	−0.262	.019	−0.124	.273
Five illustrations’ total scores of pain	0.633	<.001	0.605	<.001	−0.549	<.001	−0.337	.002	−0.188	.095
Five illustrations’ total scores of fatigue	0.307	.006	0.387	<.001	−0.257	.022	−0.372	<.001	−0.128	.258

CDAI, Clinical Disease Activity Index; HAQ-DI, Health Assessment Questionnaire Disability Index; MCS, mental component summary; MS, morning stiffness; PCS, physical component summary; QOL, quality of life; RCS, role/social summary.

Total score of the whole 15 illustrations: the total score of whole 15 illustrations for morning stiffness, pain, and fatigue.

PCS, MCS, and RCS are the summary scores of Short-Form-12.

### Correlation between the total score of all 15 illustrations and PGA and EGA scores

The total score of all 15 illustrations was significantly correlated with PGA (*r* = 0.677), EGA (*r* = 0.498), TJC (*r* = 0.549), and SJC (*r* = 0.326). In addition, PGA was also significantly correlated with the total scores of the 5 illustrations for each symptom (morning stiffness: *r* = 0.549, pain: *r* = 0.651, and fatigue: *r* = 0.353, respectively). EGA showed significant correlation with the total scores of 5 illustrations for stiffness (*r* = 0.475) and pain (*r* = 0.448), but not fatigue (*r* = 0.164) (Table 3).

**Table 3:** Correlation between the total score of the illustrations, PGA, and EGA

	PGA		EGA		TJC		SJC	
	*r*	*P*	*r*	*P*	*r*	*P*	*r*	*P*
Total score of whole 15 illustrations	0.677	<.001	0.498	<.001	0.549	<.001	0.326	.003
Five illustrations’ total scores of MS	0.549	<.001	0.475	<.001	0.394	<.001	0.259	.020
Five illustrations’ total scores of pain	0.651	<.001	0.448	<.001	0.522	<.001	0.381	<.001
Five illustrations’ total scores of fatigue	0.353	.001	0.164	.146	0.373	<.001	0.083	.465

EGA, Evaluator Global Assessment; MS, morning stiffness; PGA, patient global assessment; SJC, swollen joint count; TJC, total joint count.

Total score of the whole 15 illustrations: the total score of whole 15 illustrations for morning stiffness, pain, and fatigue.

Regarding tenderness and swelling, TJC showed significant correlations with the total scores of 5 illustrations for stiffness, pain, and fatigue (*r* = 0.394; *r* = 0.522; and *r* = 0.373, respectively). SJC was also associated with the total scores of 5 illustrations for stiffness (*r* = 0.259) and pain (*r* = 0.381), but not fatigue (*r* = 0.083) (Table 3).

### Correlation between morning stiffness duration, pain VAS, and fatigue VAS and the total score of the 5 illustrations for each symptom

Morning stiffness duration, pain VAS, and fatigue VAS showed strong positive correlations with the total score of the 5 illustrations for each symptom (*r* = 0.843, 0.802, and 0.749, respectively) and also showed correlations with each of the 5 illustrations included in each symptom (Table 4).

**Table 4:** Correlation between symptom-specific scores and corresponding illustration scores

	Illustration 1		Illustration 2		Illustration 3		Illustration 4		Illustration 5		Total score of 5 illustrations	
	*r*	*P*	*r*	*P*	*r*	*P*	*r*	*P*	*r*	*P*	*r*	*P*
Morning stiffness duration	0.860	<.001	0.512	<.001	0.527	<.001	0.495	<.001	0.530	<.001	0.843	<.001
Pain VAS	0.610	<.001	0.583	<.001	0.651	<.001	0.336	.002	0.341	.002	0.802	<.001
Fatigue VAS	0.551	<.001	0.616	<.001	0.462	<.001	0.540	<.001	0.600	<.001	0.749	<.001

VAS, visual analogue scale.

### Symptoms elicited using the illustration-based OS

Of the patients who scored 0 on morning stiffness duration (54 patients), pain VAS (26 patients), and fatigue VAS (39 patients), 5 patients for morning stiffness, 1 for pain, and 5 for fatigue had symptoms shown in the illustrations, respectively. The details of these patients are shown in Table 5.

**Table 5:** Symptoms elicited by using the illustration-based OS

								Morning stiffness duration	Illustrations of morning stiffness				
Pt No	Sex	SJC	TJC	EGA	PGA	CRP	CDAI		No. 1	No. 2	No. 3	No. 4	No. 5
1	Female	2	2	44	39	0.45	12.3	0	1	0	0	1	0
2	Female	0	0	1	3	0.09	0.4	0	1	0	0	0	0
3	Male	1	3	20	25	0	8.5	0	0	0	1	0	0
4	Male	0	0	10	8	0.05	1.8	0	2	2	2	2	2
5	Male	0	0	0	12	0.29	1.2	0	0	0	0	0	1
								Pain VAS	Illustrations of pain				
6	Female	3	0	0	10	0.1	4	0	0	1	0	0	0
								Fatigue VAS	Illustrations of fatigue				
4	Male	0	0	10	8	0.05	1.8	0	2	2	2	2	2
7	Male	2	0	0	10	0	3	0	1	0	0	0	0
8	Female	0	0	0	0	0.1	0	0	1	0	0	0	0
9	Female	0	0	0	0	0.1	0	0	1	0	0	0	0
10	Female	0	0	0	42	0.96	4.2	0	0	0	0	0	1

CDAI, Clinical Disease Activity Index; CRP, C-reactive protein; EGA, evaluator global assessment; OS, Okomarigoto Sheet; PGA, patient global assessment; SJC, swollen joint count; TJC, total joint count; VAS, visual analogue scale.

## DISCUSSION

In rheumatology practice, it is necessary for patients to communicate their conditions and opinions to healthcare professionals to facilitate SDM. However, a previous study involving interviews with patients and rheumatologists has revealed that patients often find it difficult to fully share their symptoms and communicate with doctors due to the limited time available during consultations [17]. In the current study, we demonstrated the potential of the Illustrated OS, which not only correlates with conventional measures of RA but also helps patients elicit ‘hidden symptoms’ and improve this communication, thereby supporting the practice of SDM.

Regarding disease activity and physical function, the total OS correlated with conventional disease activity and physical function evaluation indices used in daily clinical practice, such as CDAI and HAQ. These results have been confirmed in previous studies [18]. A study of patients with RA using the SF-36 also reported that RA disease activity was negatively correlated with MCS and PCS [23]. Similarly, in the present study, disease activity evaluated with CDAI was negatively correlated with PCS and MCS as assessed using SF-12. Moreover, the total score of all 15 illustrations also showed a negative correlation with MCS and PCS. Although the illustrated OS is a subjective evaluation composed of stiffness, pain, and fatigue, it was found to be associated with both physical and mental aspects. These results suggest that the total score of all 15 illustrations may have the potential to assess not only disease activity but also the physical and mental components of QOL, in a manner similar to conventional comprehensive assessments of disease activity.

In the present study, the total score of the OS showed moderate to strong positive correlations with CDAI and HAQ-DI. Because these instruments assess closely related constructs, namely disease activity and physical disability in RA, their consistent associations with the OS support the convergent validity of this measure. We also examined criterion-related validity using the SF-12 as an external standard of HL-QOL. The OS total score was significantly and negatively correlated with PCS and MCS, indicating that greater difficulties captured by the OS are associated with poorer physical and mental health status. These findings suggest that the OS reflects clinically important criteria related to impaired HL-QOL. In contrast, no significant association was found with RCS. This may indicate that the OS primarily assesses symptom- and function-related difficulties in daily life, whereas social roles and participation are more strongly influenced by psychosocial and environmental factors beyond disease activity and physical function.

Furthermore, this study suggests that fatigue in patients with RA may contribute to the discrepancy between PGA and EGA. Specifically, the total scores of the 5 illustrations for stiffness and pain were each associated with both PGA and EGA. However, the total score of the 5 illustrations for fatigue was associated with PGA but not with EGA. Moreover, previous research has indicated that EGA is more closely related to SJC than to TJC [24]. Consistent with this, our study found that SJC was associated with the total scores of the 5 illustrations for stiffness and pain, but not with the total score of the 5 illustrations for fatigue. These findings suggest that the OS is not only useful for comprehensively understanding patients' symptoms but also highlights fatigue as a significant factor contributing to the discrepancy between PGA and EGA. Many reasons have been reported for this discrepancy, and fatigue has also been identified as a potential contributing factor in previous studies [25].

This OS includes scores such as stiffness duration, pain VAS, and fatigue VAS. A high correlation was observed between each of these 3 scores and the total score of the 5 illustrations for the corresponding symptom. This indicates that the illustration-based score effectively reflects the severity of each symptom.

Additionally, in the current study, even when the evaluation of stiffness duration, pain VAS, or fatigue VAS indicates no symptoms at all (value = 0), the presence of symptoms became apparent through illustrations.

Furthermore, not only patients who met the previous stringent Boolean remission criteria but also those whose SJC, TJC, and PGA were all 0 were found to exhibit symptoms when assessed using illustrations. ‘Hidden symptoms’ that cannot be fully captured by the conventional VAS indicator may be revealed by using the illustrated OS, a visual evaluation tool that uses illustrations. A previous report has also demonstrated that using the OS many patients who achieved Simplified Disease Activity Index remission still experienced residual symptoms [26].

It has been reported that using infographics, such as illustrations, to visually display medical information allows patients to talk about things that they had difficulty putting into words, facilitating communication with medical professionals [27]. The illustrated OS is therefore considered useful not only for expressing symptoms but also for enhancing patient-doctor communication.

### Limitations

This survey has several limitations. First, this study focused solely on the group using OS, and therefore, future research should include a control group for comparison. Second, the participants were enrolled only from clinics. Therefore, patients in the hospitals should be included in further studies. Third, most patients had low disease activity. Patients with moderate to severe disease activity should be included in future studies. Fourth, we did not evaluate the test-retest reliability of the OS in this study, because patients were assessed at a single time point. Therefore, the temporal stability of the OS remains unclear, and future longitudinal studies are needed to examine its test-retest reliability and responsiveness. Fifth, we did not assess clinicians’ perspectives on the clinical utility or acceptability of the OS. Although our findings support its usefulness as a patient-reported outcome from the patients’ viewpoint, further research is required to evaluate clinicians’ acceptability of the OS, its perceived impact on clinical decision-making, and potential barriers to implementation in routine practice.

## Conclusion

The newly developed illustration-based communication tool, the OS, reflected not only the disease activity and functional disability but also the physical and mental aspects of QOL. The illustrations enabled patients to evaluate their symptoms related to RA and, moreover, to elicit ‘hidden symptoms’ that are not sufficiently captured by VAS. In addition, this survey using an illustration-based OS suggests that fatigue may be one of the reasons for the discrepancy between the PGA and EGA.

## Competing interests

MF has received speaking fees from AbbVie, Asahi Kasei, Ayumi, Boehringer Ingelheim, Chugai, Eisai, Taisho, Janssen, Mitsubishi Tanabe, Ono, and UCB and a consulting fee from Medac Japan. HN has received patent royalties from Chugai, honoraria fees from Taisho, and speaking fees from AbbVie, Ayumi, Chugai, Janssen, and Ono. TT has received research grants from Eli Lilly, Japan; consultancy fees from AbbVie, Ayumi, Bristol Myers Squibb, Eli Lilly, Gilead, Nakashima Healthforce, and Janssen; and speaker fees from AbbVie, Chugai Pharmaceutical, Eisai, Eli Lilly, Novartis, Stryker, UCB, and Zimmer Biomet. All other authors declare that they have no competing interests.
